# Eine atypische klinische Präsentation eines kongenitalen Glattmuskelhamartoms mit Alopezie

**DOI:** 10.1007/s00105-022-04957-y

**Published:** 2022-02-14

**Authors:** Chiara Giulia Schinaia, Toni Silber, Volker Beck, Katrin Kofler, Lukas Kofler

**Affiliations:** Universitätshautklinik Tübingen, Liebermeisterstr. 25, 72076 Tübingen, Deutschland

**Keywords:** Hamartom, Hypotrichie, Glattmuskelhamartom, Alopezie Becker-Nävus, Hamartoma, Hypotrichosis, Smooth muscle hamartoma, Alopecia Becker-Nevus

## Anamnese und Befund

In unserer Klinik stellten sich die Eltern eines 6 Monate alten Patienten zur Beurteilung einer auffälligen Läsion im Bereich des Kapillitiums vor. Anamnestisch zeigte sich bei Geburt ein haarloses Areal am Hinterkopf. Auch im Verlauf kam es zu keinem Haarwachstum in dem Bereich, während am restlichen Kapillitium regelrechte Behaarung vorlag. Begleiterkrankungen waren nicht bekannt.

Okzipital zeigte sich klinisch eine ca. 40 × 50 mm messende, scharf begrenzte, hautfarbene, haarlose, weiche Plaque. Aufgrund der Klinik wurde zunächst der Verdacht auf eine Aplasia cutis congenita gestellt und eine Exzision indiziert. Differenzialdiagnostisch wurde eine Atrichia congenita papulosa diskutiert. Die Eltern wünschten zunächst ein abwartendes Vorgehen, weshalb das Kind zum Zeitpunkt des chirurgischen Eingriffes bereits 2 Jahre alt war. Es erfolgten 3 Teilexzisionen in Vollnarkose zur vollständigen Entfernung des haarlosen Areals, die komplikationslos durchgeführt werden konnten. Histologisch zeigte sich im ersten histologischen Befund eine Alopezie mit miniaturisierten und abortiven Haarfollikeln. Schweiß- und Talgdrüsen fehlten fast vollständig, passend zu einer ektodermalen Fehlbildung mit Anklängen an eine Atrichia congenita papulosa. In der Befundung nach zweiter Teilexzision zeigte sich aufgrund der etwas vermehrten Mm. arrectores pili das Bild eines Bindegewebsnävus.

## Diagnose

**Figure 1:**
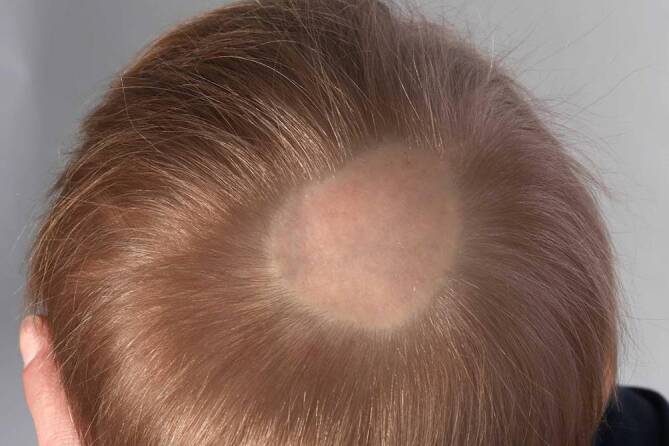

In Zusammenschau aller erhobenen Befunde konnte die Diagnose eines Glattmuskelhamartoms („smooth muscle hamartoma“) gestellt werden. Paradoxerweise wurde trotz der Lokalisation am Kapillitium keine Hypertrichose, sondern eine Alopezie hervorgerufen (Abb. 1, präoperativer Befund). Die für einen Becker-Nävus charakteristische Verbreiterung und Abflachung der Reteleisten fehlte.

## Diskussion

Glattmuskelhamartome oder Bindegewebsnävi der glatten Muskulatur sind eine seltene kongenitale, benigne und in der Regel asymptomatische Erkrankung der Haut [1, 2]. Die Erkrankung wird meist im Kleinkindesalter diagnostiziert. Klassischerweise zeigt sich das Hamartom am Rumpf oder an den proximalen Extremitäten. Glattmuskelhamartome zeichnen sich histopathologisch durch eine Proliferation der ausgereiften glatten Muskulatur der Haut (wie beispielsweise der Mm. arrectores pili, der Muskulatur der Tunica dartos am Skrotum, der glatten Gefäßmuskulatur oder der Mm. mammillae) in der superfiziellen Dermis aus. Man vermutet eine aberrante Entwicklung der glatten Muskulatur während der embryologisch mesodermalen Entwicklung im Bereich der Hautveränderung. Eine Hypertrichose im Bereich des Hamartoms wird vermutlich durch eine Stimulation der epithelialen Zellen in der Läsion durch CD34-positive dermale dendritische Zellen mediiert [3, 4]. Klinisch präsentiert sich die Erkrankung in der Regel mit einer weichen Plaque mit Hypertrichose. Das sog. Pseudo-Darier Zeichen kann bei Reibung ausgelöst werden und wird als positiv bewertet, wenn eine vorübergehende Urtika der Haut fassbar wird, welche durch Kontraktur der Mm. arrectores pilorum hervorgerufen wird [5]. Die Diagnose sollte mit einer Biopsie gesichert werden.

Aufgrund der Benignität der Läsion ist in aller Regel keine weitere Therapie erforderlich. Meist manifestiert sich ein Glattmuskelhamartom im Kleinkindesalter als solitäre Plaque mit Hypertrichose am Rumpf oder an den oberen Extremitäten.

Interessant am vorgestellten Fall sind das Fehlen der typisch klinischen Merkmale der Erkrankung mit Alopezie im Bereich der Läsion sowie das Auftreten an atypischer Lokalisation. Zudem besteht eine klinisch-histologische Divergenz bezüglich der atypischen Manifestation mit Hypotrichose und der doch typischen Merkmale in der histologischen Befundung mit vermehrten Mm. arrectores pili und weitgehender Reduktion von Hautadnexstrukturen.
